# BLE-Based Indoor Tracking System with Overlapping-Resistant IoT Solution for Tourism Applications

**DOI:** 10.3390/s21020329

**Published:** 2021-01-06

**Authors:** Radosław Belka, Roman Stanisław Deniziak, Grzegorz Łukawski, Paweł Pięta

**Affiliations:** Faculty of Electrical Engineering, Automatic Control and Computer Science, Kielce University of Technology, Al. Tysiąclecia P.P.7, 25-314 Kielce, Poland; s.deniziak@tu.kielce.pl (R.S.D.); g.lukawski@tu.kielce.pl (G.Ł.); p.pieta@tu.kielce.pl (P.P.)

**Keywords:** Internet of Things, Bluetooth, indoor tracking, mobile localization

## Abstract

In this paper, an overlapping-resistant Internet of Things (IoT) solution for a Bluetooth Low Energy (BLE)-based indoor tracking system (BLE-ITS) is presented. The BLE-ITS is a promising, inexpensive alternative to the well-known GPS. It can be used in human traffic analysis, such as indoor tourist facilities. Tourists or other customers are tagged by a unique MAC address assigned to a simple and energy-saving BLE beacon emitter. Their location is determined by a distributed and scalable network of popular Raspberry Pi microcomputers equipped with BLE and WiFi/Ethernet modules. Only simple triggered messages in the form of login records (LRs) are sent to a server, where the so-called path vectors (PVs) and interest profile (IPr) are set. The authors implemented the prototype and demonstrated its usefulness in a controlled environment. As it is shown in the paper, the solution is highly overlap-resistant and mitigates the so-called multilocation problem.

## 1. Introduction

Innovation in the tourism sector is linked to modern solutions such as Internet of Things (IoT), distributed sensor networks (DSN), cloud computing, mobile communication, and machine learning [1]. Smart tourism should provide tour information and tour guidance services before, during, and after the trip. Thus, it requires the implementation of additional solutions to support the process of their development. Two main groups of requirements for smart tourism solutions can be distinguished. The first group is related to customers (visitors). They should be able to remotely plan a specific date and route of the tour and to obtain proposed paths of seeing and basic information about expositions, as well as weather forecasts or free parking places. Moreover, tourists want to receive information about his/her current position, propositions for further routes, and additional information about a given object. An appropriate tour report including the tourist’s profile of interest and related information would also be appreciated after a finished visit.

The second group of functionalities is addressed to the management. They usually want to monitor the number of visitors in a given period, free places for given exhibition routes, and information about critical situations (overloads, emergencies, etc.). Some solutions such as automated pedestrian counters make it possible to understand how and when such facilities are being used. Unfortunately, existing solutions have many drawbacks: they are expensive, they do not provide information either about the sequence of visits (tracking functionality) or the individual preferences of the visitor, and they do not deliver personalized feedback.

This article focuses on one of the basic and very useful new functionalities, which is obtaining reliable statistical data about the degree of interest in each specific attraction—point of interest (POI)—both from a global and individual point of view. Gathering data about the level of interest in individual POIs allows park managers to better adapt the tourist offer and have more appropriate staff management. In addition, obtaining information about the individual interest of a person (so-called interest profile—IPr) significantly supports the targeting process, i.e., customizing the individual offer. For this reason, it would be expedient to develop a solution that allows anonymous estimation of profiles of interest for individual clients. This becomes possible thanks to the use of IoT solutions and a location method based on Bluetooth Low Energy technology.

## 2. IoT/BLE-Based Positioning and Tracking Systems

The Internet of Things concept is currently one of the fastest growing ICT technologies, which has a significant impact on and benefits science and the economy. These solutions are based on the idea of linking everyday objects into a computer network, mainly for the exchange, processing, and analysis of data [2]. The IoT has been functioning in global solutions for many years, although not directly under the current name. According to the IoT analytics report [3], the concept of the Internet of Things usually covers areas of the so-called smart city and connected industry and building, but also appears in the areas of smart sales or agriculture.

One of the most important components of the IoT architecture is the distributed sensor network (DSN). In the considered case, the DSN should primarily provide information about the customer’s location at a given time. There are many methods for locating and tracking a person or an object. The predominant mechanisms for tracking humans involve the use of video surveillance systems. These systems require a human operator to monitor the CCTV images at a central location. Loss of concentration usually occurs when fatigue sets in [4]. Vehicles and other objects are usually tracked using trackers whose implementation is based on the Global Positioning System (GPS). These systems display the location of a vehicle within a specified time frame. GPS, however, supports outdoor navigation since it requires line-of-sight operation with at least three satellites [5]. Another technique for implementing a tracking system is by using radio frequency identification (RFID). RFID uses either passive or active tags to track objects [6]. Passive RFID tracking is widespread in shops and libraries where tags are attached to products and are checked as they leave the shop by passing through receivers near the doors. Active RFID is popularly used in warehouses and locations such as airports where a larger range is needed. RFID tracking uses ultra-low power and there is no need for line-of-sight operation. While RFID tags are very cheap, small, and suitable for tracking objects, the sensors are considerably more expensive and require extensive configuration and software installation [7]. RFID signals are easily blocked by objects and other radio waves. One more method for tracking objects is based on GSM communication technology. The GSM equipment communicates with the GSM network through relay stations. The time at which the signals arrive, together with the angle of arrival from at least three stations, allows location detection through triangulation [8]. The main problem with GSM is the inaccuracy in location due to its limited coverage in densely populated areas [9].

The global navigation satellite system (GNSS) is the dominating technology for global positioning systems (GPSs), but since the GNSS signals are not able to penetrate buildings, other technologies should be used for indoor positioning systems (IPS). Methods using WiFi, ultra-wideband (UWB) [10], Zigbee [11], or visible light communication (VLC) were proposed. WiFi is often used for coarse urban localization with accuracy of tens of meters, but some methods for IPS were also developed [12]. The recent developments in Bluetooth technology have created new opportunities for IPS. Especially, Bluetooth Low Energy (BLE) supports low-cost, low-power beacons [13,14] that can be easily distributed. Moreover, BLE is available in all recent smartphones and many other mobile devices. However, BLE-based localization brings new challenges caused by the unique properties of BLE signals.

Indoor localization systems (ILSs) are based on measuring signals sent by transmitters. Various techniques are used for this purpose: time of arrival (TOA), time difference of arrival (TDOA), angle of arrival (AOA), and received signal strength indication (RSSI). Among them, only RSSI may be applied without additional hardware for existing wireless technologies. The most commonly used RSSI-based methods of localization are: trilateration, fingerprinting, and triangulation. Trilateration (or multilateration) [15,16,17] is based on measuring the signal strength by computing the distance between a client device and three (or more) access points with known positions. This method is simple, but since RSSI tends to fluctuate, the accuracy is low (about 2–4 m.). Fingerprinting is performed in two phases. During the first step (the offline phase), for each position the RSSI from several access points is recorded and stored in the fingerprint database. During the second step (online tracking phase), the current RSSI is compared with the values stored in the database and the closest match returns the position. This method provides acceptable accuracy (0.6–1.3 m), but in case of any changes, the database has to be updated. In the case of the triangulation method, the position is determined by measuring angles of signals received from at least three access points.

A lot of ILSs are applying the fingerprinting method. In [18], BLE was applied in offline training as well as online locating phases of the ILS. In this method, a Gaussian filter is used for the preprocessing of the signals received from BLE beacons. An ILS based on fingerprinting which uses various densities of beacons was presented in [19]. In [20], a method reducing the effort required for creating the fingerprinting map and determining the beacon positions was proposed. The method is based on graph-based optimization and uses the pedestrian dead reckoning method. Based on RSSI readings collected by a user walking in a region, the constraints for adjacent poses are generated. Then, the optimal set of poses constructing the fingerprinting map as well as the estimated positions of beacons is generated.

Since fingerprinting is known as not efficient due to the long-time location learning phase, some improvements enhancing the location accuracy were proposed. A hybrid approach based on fingerprinting and trilateration [21] uses a gradient filter for RSSI estimation. In weighted centroid localization (WCL) [22], a weight is assigned to each beacon for computing the distance. The weight is the inversed distance applied to a given degree. Thus, the beacon nearest to the location has the highest weight. In [23], a method based on fingerprinting and WCL was presented.

To achieve higher accuracy in trilateration-based methods, the inter Ring Localization Algorithm (iRingLA) was proposed [24]. Instead of one circle representing the distance from the beacon, in IRingLA, rings are used. The width of the ring corresponds to the RSSI measurement error. Then, the position is estimated as the most probable position from the intersection of at least three rings. Since the value of RSSI may depend on many factors other than the distance between devices, in [25], a method using more parameters was proposed. Instead of one instantaneous value for the RSSI/distance ratio, the reference RSSI and path lost index as well as error are computed as average values from experimental measurements of the RSSI. This way, the accuracy of 0.4 m was obtained.

Kalman-based fusion of trilateration and dead reckoning [26] enabled achieving a position accuracy of less than one meter. Dead reckoning estimates the client position assuming that the start position is known. The final position is estimated as a function of the step length and the total number of steps. An accelerometer, magnetometer, and orientation sensor are used in the localization process. A Kalman filter is used as a fusion center to compute the final position by merging positions obtained by the two fused methods. Another approach applying trilateration and Kalman filtering was proposed in [27]. In this method, the channel diversity is used to mitigate the effect of fast fading and the effect of interference during RSSI measurements. Kalman filtering is used for reducing the error caused by wrong RSSI measurements.

An example of a BLE-based localization system for smart home power management is given in [28]. The ILS is used for identification of the user location and power management using mobile devices. In [29], a system based on BLE beacons and dongles was used for user monitoring. To improve the accuracy, a machine learning algorithm was used. InLoc [30] is a system based on beacons and smartphones, which may be used for monitoring, tracking, guiding, emergency evacuation, and meeting planners. In [31], the Monte Carlo localization (MCL) method was presented. MCL is a technique for indoor localization using mobile phones with an accelerometer, compass, and BLE beacons. Some of the recent reviews of indoor localization systems and technologies are given in [32,33].

## 3. Sensor Network for Tourist Path—General Description

The preliminary concept of the system, general data structures, and some simulation analyses were presented in our previous works [34,35,36]. The presented system is primarily developed for collecting information on so-called interest profiles (activity profiles) for people visiting various types of indoor tourist attractions, such as museums or exhibitions. This means that for each client, a sequence of specific data denoted as a path vector (PV) should be obtained. The PV represents which attractions were visited, in what order, and how much interest they aroused for the visitor. 

It should be noted that individual attractions (POIs—points of interest) can be located relatively close to each other, in particular several exhibitions can be placed in one museum room. Analysis of available indoor location methods indicates that the most advantageous solution is the BLE technology. In the related literature, the basic parameter subject to optimization is the position accuracy. As mentioned, positioning techniques based on BLE can be divided into range-based method (RbM) using triangulation (trilateration), and fingerprint-based methods (FbM) using a predefined reference fingerprint map (RFM). The disadvantage of the RbM approach is primarily the unreliability and inadequacy of the propagation models used in distance estimation. The location of the signal detector and the features of the POI objects (i.e., size and electrical conductivity) have a key impact on signal attenuation. This requires individual calibration of the propagation models to effectively estimate the position. Such activities are tedious and time-consuming and indicate that an RFM would be a better approach. However, in this case, the problem may be the instability of Bluetooth emitters, which requires obtaining an RFM for each of them.

This article focuses on the most important requirement of the developed system, which is obtaining information describing the interest profile of a particular visitor. It is worth noticing that, due to the spatial size of the POI, typically from several to tens of meters, the location accuracy has low importance. This means that the appearance of a specific person near a given POI may be dichotomous. Taking into account the specificity of distributed amusement parks, in the proposed system architecture, only one scanner could be associated with one POI. The possibility of using multiple scanners is being considered to improve the system’s reliability and fault tolerance; however, this solution raises system costs. In the further part of the research, it was assumed that only one Bluetooth signal scanner/receiver is connected to one POI. Another assumption is to locate the signal detectors at a fixed position associated with the POI, while the visitors should be equipped with mobile signal transmitters. In the other proposed solutions, the role of the transmitter is often played by the client’s personal mobile device, but such a solution threatens the anonymity of visitors. In order to ensure good privacy, it is recommended to equip visitors with portable emitting devices such as dedicated battery-powered beacons. In the presented solution, beacons equipped with the CSR101x series chipset (for BT 4.1) or the CSR102x series (for BT 5.0) were proposed.

**Figure 1:**
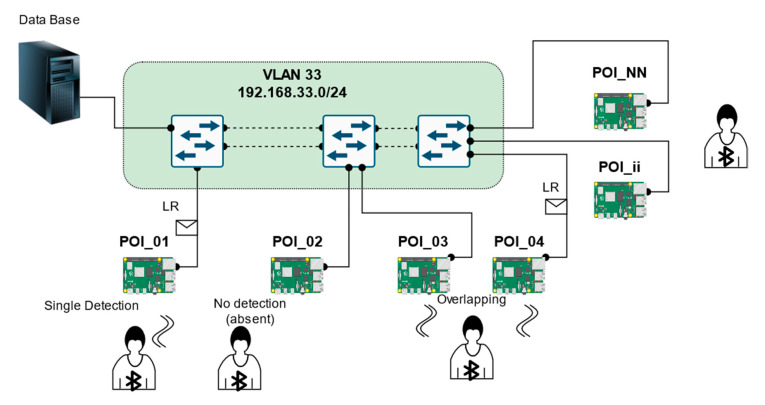
General diagram of the sensor network for a tourist path.

The scheme of the system is shown in Figure 1. The BLE signal detection can be based on a popular single-board Raspberry Pi microcomputer typically equipped with 802.15.4 BLE 4.1 modules and proper 802.3 and 802.11 network interfaces. The devices are connected to an IPv4 network capable of carrying information to the server. For the purposes of demonstration, the role of the network may be a dedicated VLAN, separated from the university network infrastructure. The prototype server was based on the influxDB solution.

Areas of the detector’s range (detection area—DA) should cover the whole POI. In typical variants known from other similar solutions, the emitters are located in fixed points near each attraction. This allows modifying the DA size using the Tx Power parameter setting option. In the proposed system, the emitters are located on the mobile client side, thus DAs will have rather comparable ranges. Meanwhile, the actual POIs in the park may be of different sizes. This will require a different approach to determining the parameter denoted as the interest level (IL), which represents the degree of interest in an attraction. Initially, this value can be related to the period of time that a visitor spent near a given attraction. Then, the key issue is to correctly recognize the moment of entry to the DA (so-called login time in—LTin) and the moment of definitive leave from the DA (LTout). However, this is not the only option. An alternative method of determining the IL parameter not directly related to the LTin and LTout timestamps will also be proposed.

The basic version of the system assumes that the individual POIs are so far apart that the corresponding DAs do not overlap. This is an assumption that may or may not be fulfilled, despite the specificity of the tourist facilities as theme parks. A client visiting individual attractions will leave behind a characteristic trace in the form of a so-called path vector (PV), which is a sequence of ILs. Basing on the PVs, information about the individual interest profile (IP) can be easily obtained. However, it should also consider situations where two or more DAs are overlapped, i.e., emitter signals are received simultaneously by two or more POI detectors. This is primarily a result of short proximity of attractions and may result in acquisition of false or uncertain statistical data. In contrast to the RbM and FbM methods, where it is a normal and desirable state, in the presented concept, it leads to an unacceptable phenomenon of multilocation. An additional assumption of the solution is to assign a given person to one and only attraction at a given time. The problem can only be slightly mitigated by adjusting the power level of Tx emitters. The goal is therefore to develop a method that can allow identifying the visitor’s position as accurately as possible. For this reason, the development and testing of a proper data processing algorithm became necessary. Implementation of the algorithm should take place on scanning devices (in the SBC RPI 4B default), and the software was further called middleware. 

## 4. Measurement Methodology

### 4.1. Experimental Procedures

The development of a prototype solution was preceded by a number of localization experiments. These experiments were divided into two phases. The main goal of the first phase was to develop a localization algorithm and choose its parameters for assuring the best localization consistency in a controlled laboratory environment. During this phase, a proprietary application for the Android 7+ operating system was used. The prototype application, called BleSkaner+, was run on smartphones. The application uses the Bluetooth 4.0+ interface for scanning nearby Bluetooth Low Energy (BLE) devices using a standard Android API [37]. For receiving as many beacon signals as possible, it uses the lowest latency mode available (ScanSettings.SCAN_MODE_LOW_LATENCY). Although the low latency mode is the most energy-consuming one, the application seems to have minimal influence on the total device battery life. For most of the devices used in the experiment, the BLE scan was automatically stopped by the operating system after about 30 min. For assuring a continuous measurement, the scan process is automatically restarted every 25 min. Research results show that the loss of signals during the restart may be neglected. The application stores scan results in CSV files in the device’s file system. These files may also be synchronized with an FTP server. The raw data were then analyzed in PTC Mathcad and free statistic software PAST 3.21 [38].

**Figure 2:**
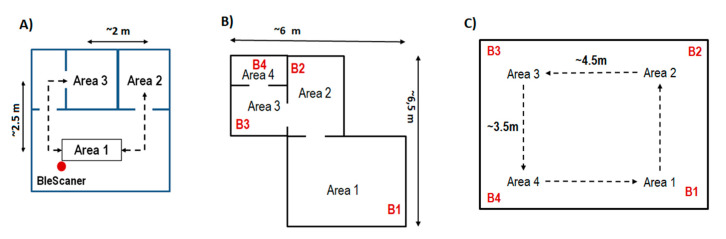
Beacon arrangement during the first phase of the experiment: (**A**) scanning process reliability; (**B**) multiroom with weak overlapping; (**C**) single room with strong overlapping.

First, basic tests of the reliability of the scanning process were carried out (A experiment). The research included the influence of beacon interval (BI) values on basic signal statistics. Then, the focus was on analyzing the position agreements. In the B and C experiments, 4 POIs (areas 1–4) and one client were included in the research model. Since the network data transmission was not fully implemented in the application, BleSkaner+ was run on the mobile side, and Bluetooth emitters (B1–B4) were stationary. Experiments were conducted in two variants: multiroom (weak overlapping) and single room (strong overlapping). Figure 2 shows the arrangement of POIs in the room.

The second phase of the experiment focused on obtaining data in an environment similar to real-life conditions. For making it possible, a prototype sensor network was built, where models of attractions (POIs) were included. Multimedia presentations displayed on 17” screen notebooks were considered as attractions. Presentations were focused on tourism and entertainment, and each was 3 to 5 min long. Initially, 12 attractions were prepared, and 9 of them were included in the experiment. The POIs were located in the halls of a two-story building, and they were separated by at least 10 m of distance. The precise location of POIs is given in Table 1. Every POI was supplemented with an Android device, which was stationary during the experiment. As previously, the BleSkaner+ application was used for scanning mobile Bluetooth beacons carried by visitors. The collected raw data were processed according to the developed algorithm and supported by the *middleware* software described in Section 4.3.

During the research, Bluetooth iNode tags were used as beacon emitters. These tags use the Qualcomm^®^ CSR1011 QFN chip and are powered by CR2032-type batteries. The tag’s beacon interval (BI) may be freely changed in a wide range from 320 ms to 10.24 s, and the Tx power of each tag is adjustable in a range from −18 to +8 dBm. For making sure the results are as precise as possible, for each tag, the Tx power was set to the maximum of +8 dB, and the BI was set to a minimal span of 0.32 s.

The model included 9 POIs and 15 different beacons. Full sequences of visiting for 32 persons were recorded. Visitors were mostly university students. One PV was not fully registered (only two of nine attractions), probably due to the emitter malfunction. Thus, the reliability of beacon emitters reached about 97.5%. Visiting time was in the range from 6 to 37 min, 13.5 min on average, and the median was 9 min. Average time spent for visiting one attraction was about 60 s.

### 4.2. Sensor Data Processing

A prototypical sensor network should be capable of estimating the interest profile data based on RSSI signals from BLE emitters. Due to the variability and unpredictability of Bluetooth signal propagation conditions, individual interpretation of the received values is required. Moreover, the radio wave interference phenomena due to the simultaneous operation of many devices result in an asynchronous and incomplete RSSI acquisition. In particular, many of the data packets sent by an emitter will not be correctly received and interpreted by a receiver. Therefore, the first step was to develop and test the aggregation and smoothing algorithm, which would compute a synchronized and smoothed waveform from the asynchronous time series of raw RSSI values. This conversion allowed the missing signal to be represented by a value of 0 instead of null. Additionally, as the RSSI value is an interval variable, we decided to convert it into a ratio variable named Vout. In other words, the input RSSI data series are smoothed and scaled to more intuitive values in the range 0–100.

**Figure 3:**
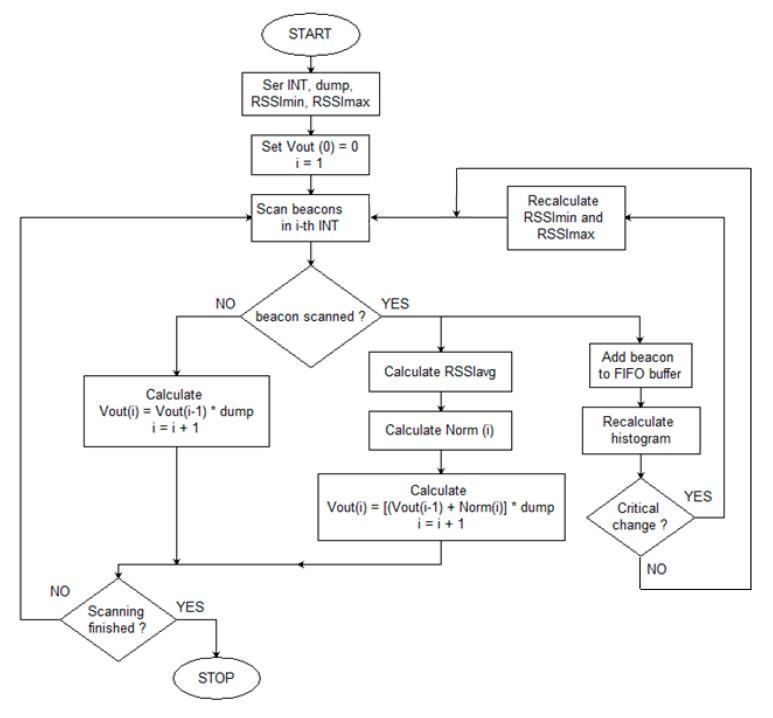
Smoothing-aggregation algorithm.

The next step is to define a decision algorithm that would qualify a moving object as present in the DA (DA presence). As a result, it became possible to obtain information about the object’s LTin and LTout. As the last element of the data processing, a special converter was designed. It accepts Vout and DA presence variables as the input and converts them to a login record whose structure was presented by the authors in [34,35]. Summarizing, the gathered data were processed according to the developed algorithm presented in Figure 3.

The variables used in the algorithm are: RSSI (in dBm), Norm(i)—scaled value for the i-th interval, and Vout(i)—output value for the i-th interval. The algorithm parameters are:

- INT—aggregation interval for a time series;
- dump—smoothing factor (blanking), number in the range 0–1;
- RSSImin and RSSImax—cut-off thresholds for RSSI values, determined based on the RSSI percentile distribution.

The algorithm is as follows:

1. Set INT, dump, RSSImin, RSSImax, and Vout (0) = 0.
2. For the given i-th INT interval, obtain the corresponding RSSI values.

If no RSSI, then calculate

(1) Vout(i)=[Vout(i−1)]·dump,

otherwise, calculate the average value of the corresponding RSSI (RSSIavg), scale it to Norm(i) by Formula (1b), and calculate the actual Vout value (1c):

(2) $$\mathrm{Norm}\left(i\right)=100\cdot \frac{1-\mathrm{dump}}{\mathrm{dump}\;}\cdot \{\begin{array}{cc} 0 & \mathrm{if}\;\mathrm{RSSIavg}\left(i\right)<\mathrm{RSSImin}\\ \frac{\text{RSSIavg}-\mathrm{RSSImin}}{\mathrm{RSSImax}-\mathrm{RSSImin}} & \mathrm{if}\;\mathrm{RSSImin}<\mathrm{RSSIavg}\left(i\right)<\mathrm{RSSImax}\\ 1 & \mathrm{if}\;\mathrm{RSSIavg}\left(i\right)>\mathrm{RSSImax} \end{array}$$

(3) Vout(i)=[Vout(i−1)+Norm(i)]·dump

and go to the next INT (increment i).

**Figure 4:**
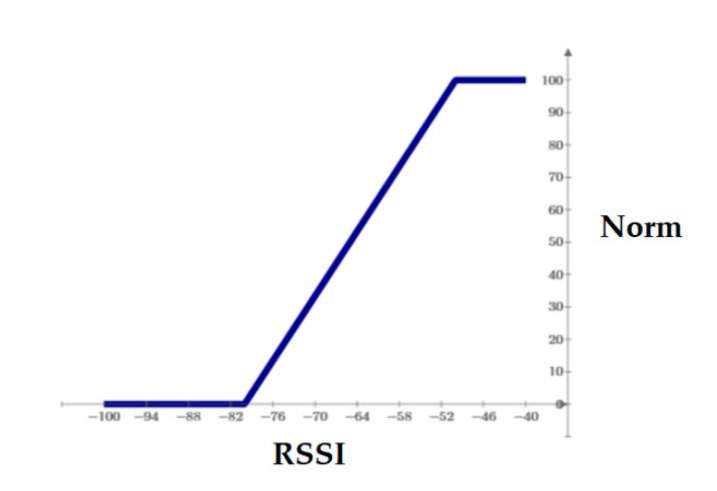
Piece-wise linear normalizing function for received signal strength indication (RSSI) to Norm transform.

The process of scaling raw RSSI values essentially came down to using a look-up table (LUT) mapping RSSI(i) values to Norm(i) values. The general linear threshold shape of the transformation function is depicted in Figure 4.

The key issue encountered while using the LUT in working conditions was the problem of setting the right values for the RSSImin and RSSImax cut-off thresholds. The assumption was made that the thresholds should represent certain values from the RSSI centile distribution. This hypothesis arose from the fact that the distribution of RSSI values usually does not have the feature of normality, which was also demonstrated by other authors [39]. For this reason, three possible approaches were considered:

- Static approach—threshold values are constant over time and identical for each emitter–scanner pair. This approach is the simplest to implement and the fastest in operation. The accuracy of the results raises doubts, given that the propagation conditions, the power of the transmitters, and the sensitivity of the receivers need not be constant.
- Dynamic approach—constant over time, but each emitter–scanner pair is assigned an individual threshold value. This approach is simple to implement and it is the most reliable; however, it can only be realized a posteriori, i.e., when all tourists have finished visiting the park. The complete state of the park would be determined, e.g., at the end of the day, which, due to the purpose of this research, turns out to be the best solution. This approach was applied to the results of phase I and phase II experiments.
- Adaptive approach (self-learning)—the threshold values are adjusted for each emitter–scanner pair during the scanning process. This approach allows self-correcting the cut-off thresholds using the historical data of recorded RSSI signals, which are stored in the form of cumulative histograms. Such histograms allow determining specific RSSI percentiles with ease.

Another important issue was the selection of the decision threshold for the Vout value, which was used to determine the tourist’s presence in the DA. In the basic variant, the discrimination algorithm assumes no overlapping. In this situation, a simple decision rule has been proposed, which tests whether the Vout signal exceeds the threshold value. The discrimination algorithm qualifies a beacon (person) as present in the DA (detection area) based on the value of Vout; in particular, it allows determining LTin and LTout information.

The proposed fixed threshold method performs simple discrimination with a threshold value proportional to max (Vout) for each emitter–scanner pair. The proposed formula is as follows:

Thresh_value = η∙max(Vout)(4)

where the η coefficient is chosen experimentally.

For the phase 1 experiment data, the decision whether an object has entered or left the DA was made when the Vout value, respectively, exceeded or dropped below the threshold of 20% of the maximum value. The disadvantage of this method is quite significant—it can be properly realized only after the end of the tourist’s sightseeing.

Characteristics of the adaptive approach applied to the aggregation-smoothing algorithm suggest that a constant Thresh_value may be sufficient because the Vout values are already normalized. However, it turns out that short-term signal loss becomes a problem because it results in unusually long intervals between two received packets. During the experiments, it was observed that the length of a critical signal break is a period of 2 to 6 s. RSSI values received after the critical break were called break-RSSI. In this case, due to the qualities of the smoothing algorithm, the Vout reaches much lower maximum values. For this reason, it becomes necessary to propose an additional correction algorithm.

Suppose that for each emitter–scanner pair, TimeBreaks stores the values of time intervals between two consecutive receiving RSSI values. By default, the values of TimeBreaks should be equal to the aggregation interval. Higher values signify disturbances in the process of obtaining location data. They should also be accompanied by rather low RSSI values, although for each scanner, the situation may be different. If higher TimeBreaks values are accompanied by stronger RSSI signals, this becomes a problem because it negatively affects Vout values. It can easily be observed that if the RSSI signal is registered every N intervals, then the Vout may approach the following limit:

(5) $$\max\left(\mathrm{Vout}\right)=\frac{\mathrm{dump}}{1-{\mathrm{dump}}^{N}}\;\cdot \max\left(\mathrm{Norm}\right)$$

Therefore, the recommended formula for calculating the decision threshold is given as follows:

(6) $$\mathrm{Thresh}\_\mathrm{value}\;=\eta \cdot \max\left(\mathrm{Vout}\right)\cdot \frac{1-\mathrm{dump}}{1-{\mathrm{dump}}^{N}}$$

The value of N can be determined adaptively based on the distribution of those TimeBreaks values that correspond to break-RSSIs. The following formula for calculating the N was proposed:

(a) Determine the median (med) of a subset of TimeBreaks values such that 1 s < TimeBreak < 6 s;
(b) Select the larger value from max (1, med −2).

If the signals are received frequently enough, then N = 1 and Formula (6) comes down to (4). In critical situations when N > 1, the decision threshold value will be lowered, compensating the potential error.

### 4.3. Prototype Middleware Implementation

Prototype middleware algorithms, described in the previous subsection, were implemented in Python programming language. In order for the application to be able to efficiently utilize multiple cores of RPi’s System on a Chip (up to four cores in the case of RPi 3B+ or RPi 4), the program logic has been divided into several processes that were designed using the consumer–producer software design pattern. The communication between processes is carried out using synchronized FIFO (First In, First Out) queues (the Queue class from the Python multiprocessing module). 

The first process, named BLEScanner, is responsible for scanning BLE devices. RSSI acquisition is performed using the commonly recognized bluepy library. However, during the middleware’s development process, one shortcoming of the library’s scanner class was discovered, and for this reason a custom scanner class derived from the default one was implemented. BLEScanner can be configured to use bluepy’s active scanning, as well as a passive mode. Furthermore, the scan’s timeout can be adjusted. Moreover, RSSI measurements can be optionally filtered by MAC addresses of the scanned devices (a list of allowed MACs can be provided).

Experiments conducted with the use of iNode tags showed that a scan timeout closely matching the advertisement time (beacon interval, BI) of tags should be avoided because in such a situation, the longest signal breaks were observed (up to several seconds). Based on the outcomes of the experiments, and because the shortest BI that can be set for iNode tags is 0.32 s, the decision was made to use active scanning with a timeout of 200 ms. This configuration provided the best results regarding the average number of acquired RSSI values per MAC per second (2.65), with relatively short and rare maximum signal breaks (1.2 s).

The second middleware process, named VoutProducer, receives raw RSSI measurements from the BLEScanner and converts them to Vout using the smoothing-aggregation algorithm described in Section 4.2:

- The aggregation interval (INT) was set to 1 s;
- The dump smoothing factor was set to 0.8 (it is configurable);
- For adjusting RSSImin and RSSImax cut-off thresholds for RSSI values, an adaptive (self-learning) approach was implemented, which uses cumulative histograms.

In order for the self-learning algorithm to adapt the cut-off thresholds dynamically and accurately even for the shortest tourist visits in POIs, it was decided to update the RSSImin and RSSImax every time 10 new RSSI values accumulated in a histogram. This computation was performed separately for each scanned BLE device because cumulative histograms are associated with individual MACs.

The third middleware process, named LRProducer, analyzes Vout signals, determines tourists’ presence in the DA, and converts Vout to login records. Vout values are aggregated individually for each BLE device in intervals named time slots (TSs). By default, a TS lasts 5 s, but its duration can be adjusted (also in real time). Time slots are synchronous—this means that each RPi calculates login records (LRs) in the same intervals. Moreover, LRProducer computes LRs only when the Vout signal reaches a certain threshold level (lower signals are discarded).

Since each of the previously described processes should be able to process (consume) some input data, as well as output (produce) some other data, to unify this behavior, an abstract ProcessWithIOQueues class was implemented. Most middleware processes are derived from this abstract class, which greatly simplifies the communication between them. Moreover, this solution also provides an easy way to configure many different consumers for data produced by a particular process, e.g., the BLEScanner can output scan data simultaneously to the VoutProducer and to some other process that transmits raw RSSI values to the central server, where they are saved in the InfluxDB database.

The nature of this research led to a strong need for conducting many comprehensive and thorough application tests. For this reason, the software was equipped with a sophisticated logging subsystem:

- Each process can log its own messages to a separate file;
- Root-level logging aggregates messages from all processes, sorts them by a timestamp using a heap queue, and outputs them to a single file (it is mainly used for reporting abnormal situations, i.e., warnings and errors);
- Messages can also be sent to syslog, which is the standard logging solution for Unix-like operating systems.

To supplement the application with the logging capabilities described above, an additional ProcessWithLogger class was implemented, which became the parent class for the ProcessWithIOQueues class. To better utilize RPi’s SoC, the final stage of logging (the interaction with the file system and syslog) is conducted in a separate process named LoggerProcess, which communicates with other middleware processes using a dedicated synchronized FIFO log queue.

The main application process is responsible for managing the middleware’s configuration, starting individual processes, and stopping them when a shutdown request is received.

## 5. Results and Discussion

### 5.1. Phase 1A Experiment—Reliability of the Scanning Process

Using the tools presented in Section 4, the RSSI values for the case shown in Figure 2A were collected. The sequence of changes was (1–2–3–2–) × 10, with 30 s presence in each area. Thus, the total scanning time was 4 × 10 × 30 s = 20 min, whereby 25% of the total time was spent in AREAs 2 and 3 and 50% in AREA 1. The measurement was repeated for three different BIs: 0.32 s (2690 analyzed RSSI values), 1.28 s (672 analyzed RSSI values), and 5.12 s (180 analyzed RSSI values). Basic statistical measures were calculated, along with the distribution of numerical data (histograms). The base for the analysis was primarily RSSI values in the typical range: −100 to −30 dBm. Moreover, unexpected interruptions (so-called breakTime) were taken into account. Interruption was assumed critical if its time span was longer than ceil(3∙BI).

Table 2 presents three representative parameters of a set of RSSI values: packet processing efficiency (PPE), RSSI median, and RSSI interquartile range (IQR). PPE is computed as a ratio of the number of received packets to the theoretical number of packets sent by a packet emitter, resulting from the presupposed BI. Due to the observation that the distribution of data was skewed instead of normal, median and IQR statistical measures were used.

**Figure 5:**
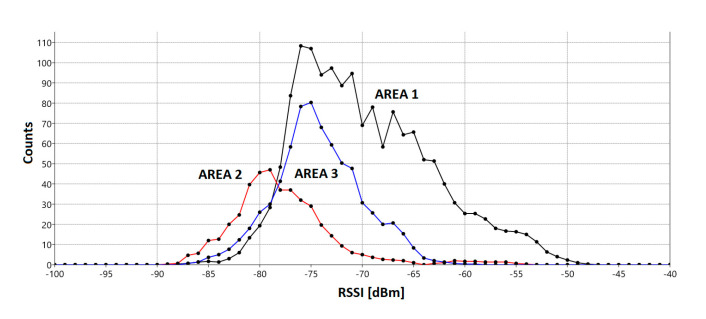

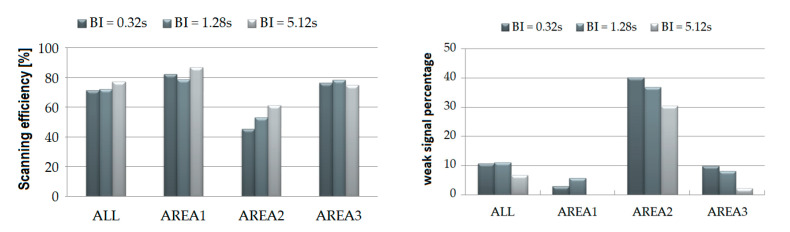
Statistical parameters of Bluetooth Low Energy (BLE) signals for different values of the beacon interval.

The PPE reliability factor reached 70% and more, which means that, e.g., for BI = 0.32 s, ~two independent signal strength measurements per second are anticipated. As expected, the average signal strength for AREA 1 was the highest, as was the PPE. In AREAs 2 and 3, more but comparably separated, the signal strength is noticeably weaker. Particularly, it was noticed that the signal from AREA 2 is weaker due to a metal locker placed in the room, which reduced propagation of the signal. Different signal parameters are reflected in the distribution (histograms) of the measurements, with a decrease in PPE from 80% to a mere 45% and an exceptionally high rate of critically weak signals (RSSI < −80 dBm), as shown in Figure 5.

The results show a slight improvement in the signal quality for longer beacon intervals. This indicates that in less dynamic scenarios, the BI could be increased, not only for the sake of energy savings, but also due to the improved reliability of the data transmission.

With respect to the *breakTime,* it was verified that for BI = 0.32 s, less than 2% of received packets were preceded by critical interruptions longer than 1 s, whereby the maximum interruption was 3 s for AREAs 1 and 3 and up to 8 se for AREA 2. For BI = 1.28, the share of critical interruptions (>4 s) was about 4.4%, whereby the maximum interruption was 12 s for AREAs 1 and 3 and up to 27 s for AREA 2. For BI = 5.12 s, no maximum interruption was longer than 20 s, whereby about 3.3% lasted longer than 15 s.

### 5.2. Phase 1B Experiment—Position Agreement Test for Multiroom Weak Overlapping Case

In this part of the experiments, the RSSI values for the case shown in Figure 2B were recorded. The sequence of changes was (1–2–3–4–3–2–) × 5, with 30 s presence in each area. Thus, the total scanning time was 5 × 6 × 30 s = 15 min, whereby ~16.7% of the total time was spent in AREAs 1 and 4 and ~33.3% in AREAs 2 and 3.

**Figure 6:**
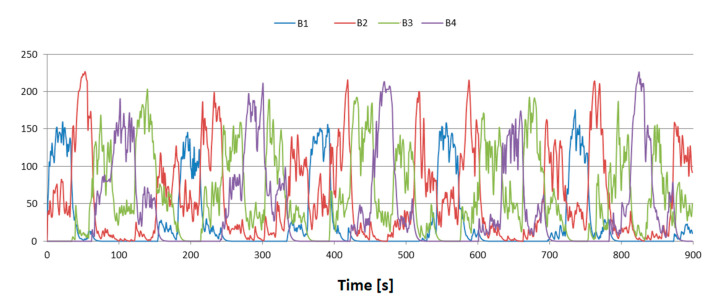
Processed Vout time series.

Figure 6 shows the processed time series. The values INT = 1 s and dump = 0.65 were adopted, while RSSImin and RSSImax were estimated from the percentile distribution of RSSI values for levels 0.4 and 0.95, respectively.

Next, the processed data were classified according to the “Winner Takes All” (WTA) principle. This means that the classification was based only on the highest Vout value criterion. An additional condition was introduced—the ratio of the highest Vout value to the second one must exceed the given threshold *h*. Otherwise, the algorithm will return 0, indicating that the person is outside of any DA. The algorithm is as follows:

1. For a given aggregation interval INT, create a so-called fingerprint vector (FV) containing the corresponding Vout values;
2. Sort the FV in descending order -> FVsort;
3. Check if (FVsort [0] < Thresh_value) or (FVsort[0]/FVsort[1] < h):
  if the condition is true, then positions [INT] = 0;
  otherwise, positions [INT] = index + 1, where the index should satisfy formula: FV [index] = FVsort [0].

**Figure 7:**
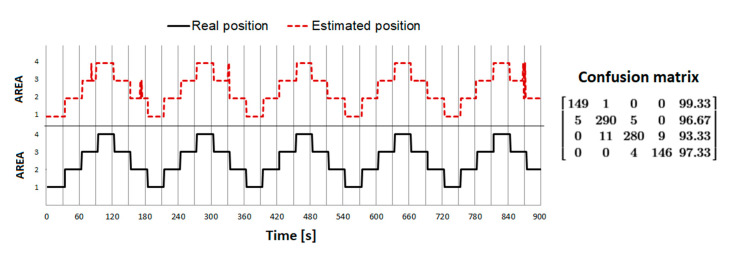
Graph showing the real position (solid line) vs. the estimated decision function (DF—dashed line) determined in phase 1B experiments using the proposed algorithms.

The result of the algorithm is a time-dependent relationship representing the belonging of a given person to a particular DA. This time series has been named the decision function (DF). It is assumed below that individual POIs are identified by natural numbers from 1, whereas 0 denotes a position outside of any DA. The results are shown in Figure 7. The h value was assumed to be 1.

In order to objectively estimate the accuracy of the positioning, the following numerical measures were proposed:

- Position agreement (PA)—percentage of compliance of relevant values in real position and DF time series;
- Visit period error (VP error)—the relative difference between the real and estimated time spent in the area.

The highest PA, ~96.1%, was obtained for dump = 0.65 and low_perc = 0.4. As expected in the extreme positions (Area 1 and Area 4), the PA is greater, in the order of 98–99%. In addition, a location error often occurs during crossing area boundaries, which is a normal and acceptable situation. The VP error for individual positions was 2.7% for AREA1, 0.7% for AREA2, 3.7% for AREA3, and 3.3% for AREA4, and the total average error was ~ 2.4%.

### 5.3. Phase 1C Experiment—Position Agreement Test for Single Room Strong Overlaping Case

**Figure 8:**
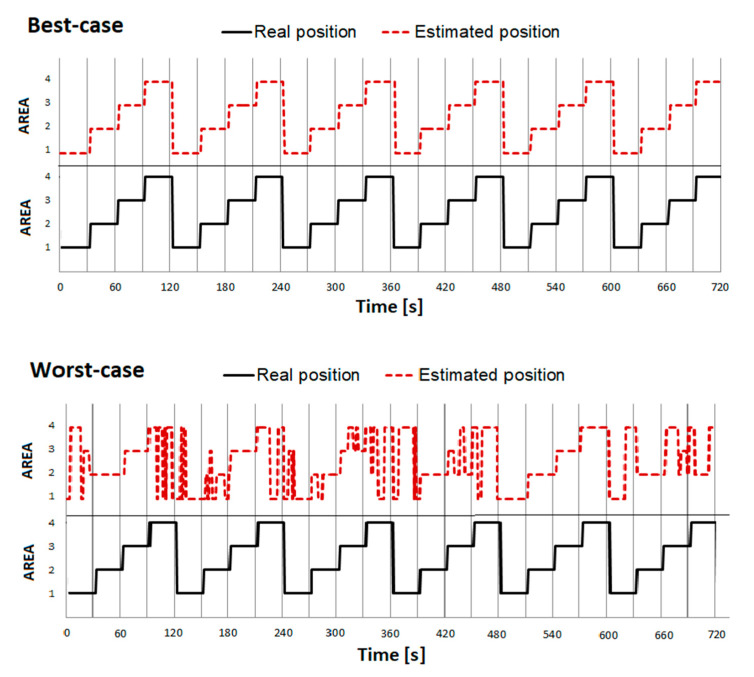
Graph showing the real position (solid line) vs. estimated decision function (DF) (dashed line) determined in phase 1C experiments using the proposed algorithms.

In this phase, the RSSI values for the case shown in Figure 2C were recorded. The sequence of changes was (1–2–3–4–) × 6, with 30 s presence in each area. Thus, the total scanning time was 4 × 6 × 30 s = 12 min, whereby ~25% of the total time was spent in AREAs 1–4. In addition, the experiment was carried out in two variants: the more favorable—back to the room, facing the emitter (best case), and the less favorable—front facing the room, back to the emitter (worst case). This is due to the fact that the beacon emitter was placed in the form of a necklace, located at the front at the level of the chest. It was to be expected that the propagation conditions along the emitter–scanner path would be different, mainly due to the wave absorption by the human body. This way, the effect of either the translational or rotational position was studied. Similarly, to experiment B, the position agreement was determined—Figure 8.

Despite the relatively close location of the emitters and strong overlapping, the best-case variant achieved the PA value at 97.9%. However, in the worst-case variant, as expected, the compliance was unacceptably low and estimated to a maximum of ~ 73%. The average RSSI values recorded from the emitter, appropriate for a given position in individual positions for best-case and worst-case scenarios, were also compared. The differences were from 4 to even 10 dBm, depending on a particular location. This points to the unreliability of FRM-based localization methods, suggesting the need for a higher number of detectors.

### 5.4. Discriminant Analysis vs. WTA Approach

**Figure 9:**
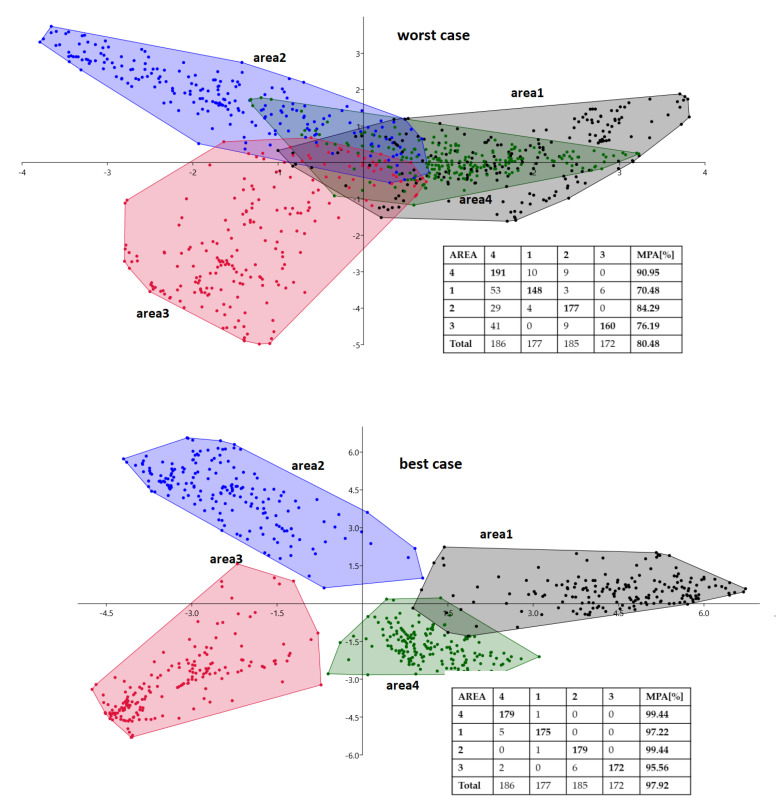
Linear discriminant analysis using the CVA method for worst-case and best-case variants of phase 1C experiments (confusion matrices included).

An effective way of assessing the ability to discriminate profiles is to perform a linear discriminant analysis (LDA). The objective of the LDA is to estimate discriminant functions that are a linear combination of independent variables that will discriminate between the categories of the dependent variable in the most effective manner. It also evaluates the accuracy of the classification. In the considered case, the canonical variate analysis (CVA) method was used [38]. Results for phase 1C experiments are presented in Figure 9.

The number of samples in the considered case was 720 and corresponded to the signal measurements for every second from four scanners. The classes correspond to four locations (scanners) and there should be 180 observations in each of them. As shown in the inset table in Figure 9, the number of classified samples presented in a diagonal position for each class slightly differs from 180.

In the best-case scenario, the discriminatory effectiveness of the CVA method and the method based on the WTA criterion was similar. On the other hand, in the worst-case scenario, the discriminatory effectiveness was theoretically improved from around 73% to around 80%, which unfortunately is still low. This means that when all four values are taken into account during the discrimination analysis, the system’s locational capability can only improve slightly. Moreover, the conducted experiments led to an interesting observation. People who were close to an attraction, but were facing the opposite direction, were more often qualified as absent (position 0). This may have a positive impact on the resulting statistics of interest profile measurements.

### 5.5. Phase 2 Experiment—Results

During the experiment, a total of 96,502 packets were analyzed, with an average of 715 per minute. Only one emitter out of 32 was temporarily inactive (for seven out of nine POIs). The reliability level has been estimated at 97.5%, which is acceptable from a practical point of view. Median value of beacons registered for one client for one POI was 182, interquartile range: (IQ: 97—356). On average, the system processed approximately three beacons/s, which is a similar value to the number of beacons sent by one emitter. An important element of the solution developed in phase 2 (middleware) is the Vout and DA presence converter to record login, as conversion to an LR is an important element of the IoT architecture. A login record is a structured unit of information, representing one event, i.e., the presence of an emitter (tourist) in a given DA (attraction). During the experiment, a total of 1093 LRs were registered, i.e., on average ~ 34 per visitor. About 52% of LRs had a time shorter than 5 s. On the other hand, the number of LRs with a VT of 10 s or more was 379 (~ 12 per visitor). In further analysis, only LRs with a time of 5 s or longer were accepted. The average number of accepted LRs generated by one scanner was 1.44 per minute, where for visitors who devoted more than 1 minute to one attraction (11 people known as long-term visitors), this parameter was 1.2 per minute, while for the other visitors (21 people known as short-term visitors), this parameter was 1.57 per minute. It can be assumed that in a situation where ~20 people would be present at the same time in the DA, the related scanner will generate an average of ~ 30 LRs per minute, which should enable their smooth transfer to the central server even when using low-bandwidth technology such as LoRaWAN.

**Figure 10:**
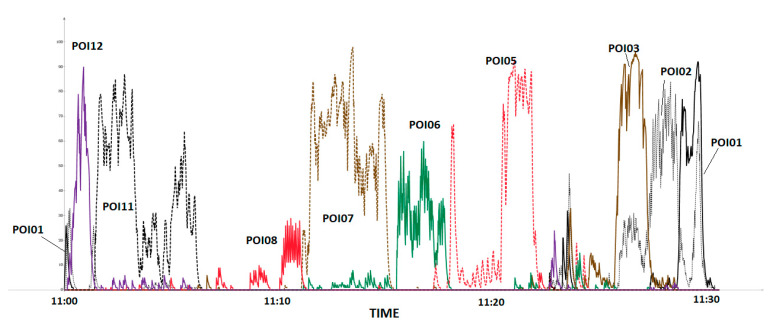
Vout time series for a representative visitor.

An example of the Vout time series for one of the visitors is shown in Figure 10. There is a clear signal overlap which leads to the problem of multilocation.

The overlapping factor has been introduced as the ratio of the overlap time to the total visiting time. This coefficient was determined for each visitor independently, in two variants:

- Vout overlapping (Voverlap)—a situation where the Vout value is nonzero for at least two scanners simultaneously. It is an indicator of the physical signal overlap;
- LR overlapping (LRoverlap)—a situation where the time intervals of two or more LRs collide with each other, interpreted as an unacceptable multilocation situation (being simultaneously in two or more DAs).

First of all, it is advised to minimize LRoverlap equal to 0, while Voverlap can be greater than 0. Note that in the absence of the proposed analytical solutions, the LRoverlap value is equal to Voverlap. The average value of the Voverlap coefficient for 32 PVs was approximately 68% (mean = 0.6826, stdev = 0.1632). This means that only in ~ 32% of the visit time would the location be unambiguous. Moreover, the degree of multilocation for Voverlap was found to be 3 to 7 (i.e., in some cases, a maximum of seven scanners simultaneously generated a nonzero Vout signal). Such low efficiency is unacceptable from a practical point of view.

Thanks to the implementation of middleware algorithms, the average LRoverlap value was clearly lower: average ~ 0.1455, stdev = 0.0595, max 0.375. This means that, on average, the visitor’s location is practically undefined due to multilocation only for ~1/7 of the visiting time. Furthermore, the degree of multilocation was not greater than 3.

The research also suggests that the value of the overlapping factor depends on the time of the visit. The average Voverlap values turned out to be slightly lower (~0.6) for long-time visitors compared to short-time visitors (~0.72); however, the differences are at the border of statistical significance (*t*-test for CI = 0.95, p ~ 5.9%). A stronger difference was observed for LRoverlap, where 0.115 vs. 0.161 values were estimated, respectively.

### 5.6. IoT Architecture vs. LR Overlapping

As mentioned above, a login record (LR) is a structured unit of information representing the event characterized by the presence of a particular emitter (tourist) in a given DA (attraction). The Vout to LR conversion performed by the scanner is optional, as it is possible to send Vout data directly to the central server. However, sending just login records can significantly reduce the amount of information being transmitted. In the basic version of the system, it is required to send one information frame per second per MAC. Meanwhile, the conducted experiments demonstrated that the average number of information frames sent by a scanner can be as low as 1.5 frames per minute per MAC, which is about 40 times less than the theoretical number. This observation makes the idea of using dedicated network technologies such as LoRaWAN very plausible.

Each login record contains the following information:

- POI (point of interest, attraction) identifier;
- Beacon (tourist) identifier (MAC, beaconId, etc.);
- The moment in time of entering the DA (LTin, short for login time in);
- Visit time (VP, short for visit period) or equivalent information about the moment at the time of leaving the DA (LTout, short for login time out), where VP = LTout-LTin+1;
- Additionally, the level of tourist interest in the attraction (IL, short for interest level).

The value of the IL parameter is calculated as the Vout cumulative value in a period of time determined by the LTin and LTout timestamps. To be more precise, this calculation requires knowledge of the discriminant function corresponding to the Vout values, and then the whole formula can be written as follows:

(7) $$IL\left(POI\right)=\;\int_{T_{IN}}^{T_{OUT}}Vout\left(t\right)\cdot \left[DF\left(t\right)=POI\_Id\right]\cdot dt$$

It should be noted that IL can also be represented as information about the average signal strength. However, the original representation as cumulative signal strength allows a simple aggregation of LRs in a situation when a particular tourist has visited a given DA several times.

Login records can be synchronous or asynchronous. Synchronous LRs are calculated in specific moments in time, e.g., once every 5 s, 10 s, or 30 s. Additionally, an LR will not be sent by the scanner if the Vout value is below the decision threshold level, which makes it easier to tackle the problem of overlapping. Asynchronous LRs are sent the moment the LTin and LTout parameters have been determined—this approach was used during the research stage. However, with asynchronous LRs, there is always a risk of system starvation when the VP is very long. In such a situation, it is recommended to segment the LRs into shorter fragments (periodic LRs).

In the context of the topics discussed so far, a certain issue of great practical significance emerges—the problem of multilocation. The simplest solution to the overlapping problem involves comparing the value of Vout signals and choosing the strongest one at any moment in time. Based on this information, a decision about the tourist current location should be made, as was done during the phase 1 experiments. However, it requires a centralized approach which necessitates sending raw RSSI data, or at least Vout values, to the server where the decision will be determined. This solution reduces beacon scanners to the role of signal retransmitters and is not conformable to the concept of edge computing and IoT.

The IoT approach assumes that the server receives data only in the form of LRs, i.e., without the Vout values. Despite that, the authors devised a solution to the overlapping problem. As a comparative criterion, a simple parameter was proposed, calculated as the ratio of IL to VP, representing the average value of Vout. The analysis of data acquired during the experiment allowed comparing both methods and assessing the consistency of the results produced by the IoT approach with the results computed by the theoretically more effective centralized approach. The average effectiveness of the IoT approach (IoT agree) compared to the central one was about 96.7 ± 1.8%. For long-term visitors, the effectiveness was even higher—about 98 ± 1%. This means that the decisions made only on the basis of LRs data are almost identical to those determined with the centralized approach. Detail information for all 32 visitors is presented in Table 3. 

The basic innovation of the described solution concerns transferring a significant part of the decision-making process from the central server to intermediate devices—IoT gateways, built on the basis of SBCs (Single Board Computers). In the proposed solution, a set of special packets called LRs is sent instead raw packets. The most visible advantage concerns increasing the involvement of intermediary scanning units in the location detection process, limiting data transfer to the most important ones (see scanned beacons/min vs. LR density/min in Table 3), relieving the central server and database, and making the architecture more flexible. At the same time, as shown in the IoT_agree parameter in Table 3, the level of localization accuracy is only from ~1 to ~5% worse than in traditional, centralized solutions. It should also be noted that the IoT approach in its current state does not require communication between RPis. This makes the proposed solution less complex and, at the same time, satisfactorily effective.

## 6. Conclusions

This article presents the concept and pilot implementation of an indoor tracking system, whose main purpose is acquiring relevant information about the behavior of visitors. The concept of this work was to create a tourist service system, but it may be more widely used. The precise position of a person is not so important in this case (unlike in GPS-like systems), but rather the interest level of a person for each POI is, which is represented by an integrated value of a properly scaled RSSI rather than the duration of the visit. Such an approach makes the process of acquiring the most important data concerning a visit easier. The problem, however, is the strong overlapping of signals, especially in the case of short proximity to other POIs. This leads to the multilocation problem, which makes neither the proper recognition of a path nor computing the correct values of the interest level parameter possible. In the paper, a solution to this problem was proposed, where properly prepared signals in individual time windows are compared and the WTA (Winner Takes All) principle is applied. Conducted experiments show that such an approach gives satisfactory results.

Currently, the proposed system is run as a distributed sensory network built on the basis of popular Raspberry Pi 4B SBCs. Raw RSSI data are retrieved with the help of the widely known bluepy library and then it can be processed using the middleware presented in this article. It is intended that the output in the form of integrated LR packets is delivered to the InfluxDB database system, dedicated to retrieving and processing time series. In the centralized system, decisions concerning the identification of location and computing IL parameters for each POI are taken. Further plans include creating software for collecting ILs sequences (so-called behavior vector) and their statistical analysis. It is worth mentioning that the architecture of the proposed solution allows for integration of the system with alternative solutions using, e.g., tracking with the use of GPS technology.

## Figures and Tables

**Table 1:** 3D position of points of interest (POIs) (attractions) used in the second stage of the experiment. The number of stair steps is used for the Z coordinate.

POI	X [m]	Y [m]	Z [step]
START/FINISH	0	24	27
01	1	14	27
02	0	2	30
03	10	0	30
05	32	1	30
06	46	1	30
07	51	0	0
08	39	0	0
11	4	0	0
12	2	10	3

**Table 2:** Basic statistical parameters of “A” scanning experiment.

	BI [s]	ALL	AREA1	AREA2	AREA3
**PPE. [%]**	0.32	71.72	82.51	45.44	76.48
	1.28	72.27	78.93	53.19	78.51
	5.12	77.57	87.07	61.44	75.09
**Median**	0.32	−73	−71	−79	−75
	1.28	−73	−71	−76	−74
	5.12	−72	−66	−77	−72
**IQR**	0.32	8	10	5	5
	1.28	9	10	7	6
	5.12	12	13.5	6.5	5

**Table 3:** Voverlap vs. LRoverlap values for observed visitors—details.

Client_Id	Total Visit Time [min]	Scanned Beacons /min	Number of LRs	LR Density [LR/min]	Vout Overlap [%]	MaxOverlap for Vout	LR oVerlapping [%]	IoT_Agree [%]
C01	10	270.5	11	1.10	74.33	5	12.00	97.33
C02	9	243.1	12	1.33	72.22	4	14.81	95.19
C03	12	192.6	18	1.50	81.25	5	15.97	97.36
C04	8	323.1	10	1.25	92.08	5	15.83	96.25
C05	9	53.9	17	1.89	33.89	3	14.26	99.63
C06	7	214.4	9	1.29	72.86	4	14.76	96.19
C07	8	172.8	11	1.38	53.96	5	13.33	97.71
C08	7	207.3	11	1.57	65.24	5	14.76	94.29
C09	8	205.1	14	1.75	73.75	5	13.75	97.50
C10	6	227.3	11	1.83	77.50	4	17.50	94.72
C11	9	291.2	13	1.44	81.85	7	21.67	96.30
C12	8	222.4	15	1.88	71.04	6	17.92	93.75
C13	8	174.1	13	1.63	76.67	5	13.33	95.83
C14	8	259.1	10	1.25	78.33	5	14.38	95.63
C15	8	228.1	13	1.63	68.54	5	11.04	97.08
C16	25	264.4	23	0.92	77.67	5	9.87	97.87
C17	30	235.3	28	0.93	52.44	5	10.89	98.61
C18	12	234.0	32	2.67	80.69	6	37.50	90.83
C19	8	294.1	15	1.88	79.79	5	18.33	95.42
C20	8	237.1	11	1.38	79.58	4	15.42	95.21
C21	7	163.1	14	2.00	48.57	3	20.48	96.43
C22	8	234.6	11	1.38	77.92	4	12.92	97.29
C23	13	186.8	24	1.85	72.14	3	23.33	97.18
C24	18	232.1	32	1.78	65.93	5	18.61	95.65
C25	31	257.7	21	0.68	58.76	4	7.47	99.14
C26	37	75.4	60	1.62	27.03	3	13.15	97.57
C27	15	200.4	17	1.13	76.67	5	11.11	97.22
C28	15	217.5	20	1.33	64.11	5	9.00	98.44
C29	14	167.4	13	0.93	28.45	4	3.57	98.69
C30	12	257.4	12	1.00	75.69	5	9.03	97.22
C31	28	318.9	25	0.89	92.08	5	9.64	98.99
C32	25	315.0	29	1.16	53.13	6	9.93	98.27

## Data Availability

Data is contained within the article.
